# Thin metal nanostructures: synthesis, properties and applications

**DOI:** 10.1039/c4sc02571g

**Published:** 2014-09-23

**Authors:** Zhanxi Fan, Xiao Huang, Chaoliang Tan, Hua Zhang

**Affiliations:** a School of Materials Science and Engineering , Nanyang Technological University , 50 Nanyang Avenue , Singapore 639798 , Singapore . Email: hzhang@ntu.edu.sg ; http://www.ntu.edu.sg/home/hzhang/ ; Fax: +65 67909081

## Abstract

Two-dimensional nanomaterials, especially graphene and single- or few-layer transition metal dichalcogenide nanosheets, have attracted great research interest in recent years due to their distinctive physical, chemical and electronic properties as well as their great potentials for a broad range of applications. Recently, great efforts have also been devoted to the controlled synthesis of thin nanostructures of metals, one of the most studied traditional materials, for various applications. In this minireview, we review the recent progress in the synthesis and applications of thin metal nanostructures with a focus on metal nanoplates and nanosheets. First of all, various methods for the synthesis of metal nanoplates and nanosheets are summarized. After a brief introduction of their properties, some applications of metal nanoplates and nanosheets, such as catalysis, surface enhanced Raman scattering (SERS), sensing and near-infrared photothermal therapy are described.

## Abstract 2

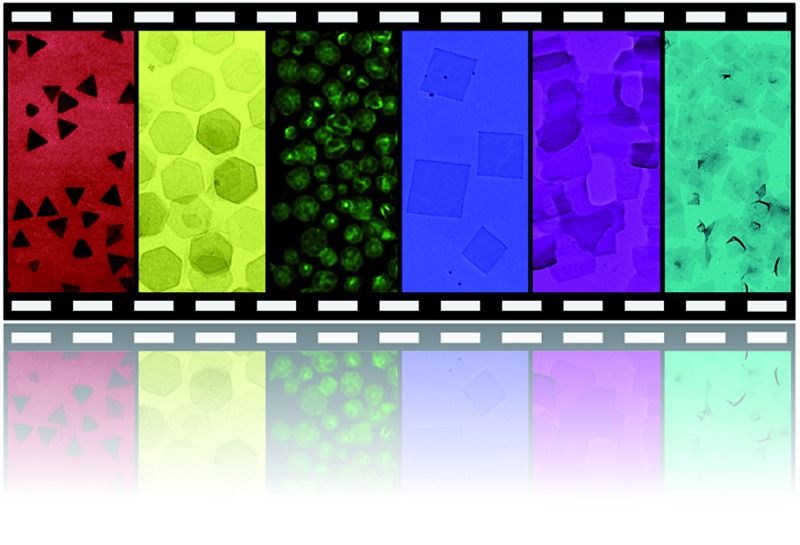
This minireview introduces the recent progress in the synthesis, properties and applications of thin metal nanostructures, especially metal nanoplates and nanosheets.

## 1. Introduction

In addition to the compositions, crystal phases and morphologies, the essential role of dimensionality in determining the intrinsic properties and functions of nanomaterials has been highlighted since the observation of graphene in 2004.^1–3^ Graphene, a single-layer carbon sheet, has shown many unconventional physical, chemical and electronic properties arising from its two-dimensional (2D) structure.^4–8^ Inspired by graphene, other kinds of 2D nanomaterials, such as transition metal dichalcogenide (TMD), transition metal oxide/sulfide and boron nitride nanosheets, are also receiving extraordinary research interest.^9–20^ The unique physical, chemical and electronic properties of these 2D nanostructures make them promising candidates for a wide range of applications, such as in electronic devices, catalysis reactions, sensing and energy conversion and storage devices.^11,17,18,20–26^ Importantly, the rapid development of 2D nanomaterials has stimulated research in the controlled synthesis of thin nanostructures of some traditional materials (*e.g.* metals) and the exploration of their potential applications.^27–34^

Metals are a class of materials that have a long history in the fundamental studies and industrial applications.^35,36^ It is well known that the properties and functions of metal nanomaterials are closely correlated with their size, shape, structure and composition. Therefore, the controlled synthesis of metal nanocrystals for various applications has been extensively studied in the last two decades.^37^ Recently, great efforts have been devoted to the controlled synthesis of thin nanostructures of metals, especially metal nanoplates and nanosheets. Similar to graphene and its inorganic analogues, metal nanoplates and nanosheets have also shown some unique properties compared to nanostructures with other shapes, such as nanoparticles, nanorods and nanowires.^27–32,34,38–42^ Taking Au as an example, while the absorption of Au spherical nanoparticles mainly lies in the ultraviolet-visible (UV-vis) region, Au nanoprisms show strong near infrared (NIR) absorption, thus making them promising candidates for NIR-related applications (*e.g.* NIR photothermal therapy).^39,42–45^ In addition, the sharp corners and edges of Au nanoprisms exhibit particularly strong localized surface plasmon resonance (LSPR), which favors their plasmonic applications (*e.g.* surface enhanced Raman scattering, SERS).^37,46^ Moreover, ultrathin Au square sheets (AuSSs) with a thickness of 2.4 nm are found to crystallize in the hexagonal close-packed (hcp) structure instead of the face-centered cubic (fcc) structure commonly observed in spherical Au nanoparticles, nanorods and nanowires.^28,37,47^ In this minireview, the recent progress in the synthesis and applications of metal nanoplates and nanosheets is reviewed. After introducing the synthetic methods and properties of metal nanoplates and nanosheets, their applications in catalysis, SERS, LSPR-based sensing and NIR photothermal therapy are demonstrated.

## 2. Synthetic methods

It is well known that the structures of graphite and bulk crystals of TMDs are layer-structured materials, in which the layers stack together *via* weak van der Waals forces. Therefore, graphene and single- or few-layer nanosheets of TMDs can be directly prepared by exfoliation of their bulk crystals, *i.e.* top-down methods, such as mechanical exfoliation, chemical/electrochemical Li intercalation and exfoliation, and solvent-assisted exfoliation.^3,8,11,15–17,23,48–50^ Conversely, metal atoms bond together *via* strong metallic bonding. Therefore, it is very difficult to prepare metal nanoplates and nanosheets by directly exfoliating their bulk crystals. To date, many methods have been developed for the synthesis of metal nanoplates and nanosheets and most of these methods belong to the bottom-up synthesis category. The bottom-up methods, which start from metal salts or small metal nanoparticles, include surfactant-mediated synthesis,^51,52^ polyol reduction,^40,53,54^ 2D template-assisted synthesis,^28,41,55^ seeded growth,^39,56–58^ photochemical synthesis,^30,31,59^ ultrasonic methods,^60^ hydrothermal and solvothermal methods,^27,34,61^ carbon monoxide and halide ion confined growth,^29,62^ biological synthesis,^42,63–65^ and self-assembly of nanoparticles.^66,67^ Besides the aforementioned bottom-up synthesis, the top-down approaches, such as electron beam nanolithography, nanoimprint lithography and hole–mask colloidal lithography,^68–70^ have also been developed to prepare metal nanoplates and nanosheets. In this section, we will mainly focus on the introduction of some bottom-up approaches (Table 1).

**Table 1:** Summary of the synthetic methods discussed here for preparation of thin metal nanostructures

Synthetic methods	Metals	Structures	Morphology	References
Surfactant-mediated synthesis	Pt, Au–Pd, Cu, Ag, Pd, Rh, Au, Ni–Cu	fcc	Nanowheel, nanodisk, nanoplate, nanosheet	47, 51, 52, 73, 75, 84, 89 and 91
	Co	hcp	nanodisk	32
2D template-assisted synthesis	Au, Pd, Pt, Ag	fcc	Nanosheet, nanoplate	33, 55, 95 and 98
	Au	hcp	Nanosheet	28
Seeded growth	Ag, Au, Au@Ag, Au@Cu, Au@Ni, Pd@Ag, Pd@Pt, Ag@Au	fcc	Nanoplate, nanoprism	39, 57, 58, 73, 104, 107, 108 and 110
	Au	hcp/fcc	Nanoplate	56
Photochemical synthesis	Ag, Au@Ag, Au	fcc	Nanoprism, nanoplate	31, 59 and 121
Hydrothermal and solvothermal methods	Ag, Pt–Cu	fcc	Nanoplate	123 and 34
	Ru, Rh, Bi–Rh	hcp	Nanoplate, nanosheet	27, 61 and 127
Carbon monoxide and halide ion confined growth	Pd, Au	fcc	Nanosheet, nanoprism	29 and 137
	Pt–Bi	hcp	Nanoplatelet	138
Self-assembly of nanoparticles	Au	fcc	Nanosheet	67

### 2.1. Surfactant-mediated synthesis

Generally, the surfactant-mediated synthesis method used for metal nanoplates and nanosheets is to reduce the metal salts by using a reducing agent assisted by the surfactant. This method allows one to easily prepare metal nanoplates and nanosheets in one pot at low cost. So far, surfactants used for the synthesis of metal nanoplates and nanosheets can be classified to the following groups based on their properties: (1) ionic surfactants, such as cetyltrimethylammonium bromide (CTAB),^51,71–74^ octadecyltrimethylammonium chloride (OTAC),^52^ sodium di(2-ethyl-hexyl)sulfosuccinate (Na(AOT))^75–77^ and sodium dodecylsulfate (SDS);^78^ (2) polymer surfactants, such as poly(vinyl pyrrolidone) (PVP),^79–86^ poly(vinyl alcohol) (PVA)^87^ and poly(ethylene oxide)–poly(propylene oxide)–poly(ethylene oxide) (P123);^47^ (3) nonionic surfactants, such as oleic acid (OA),^32,88^ oleylamine (OAM),^89,90^ trioctylphosphine (TOP)^91^ and trioctylphosphine oxide (TOPO).^32,88^

**Fig. 1:**
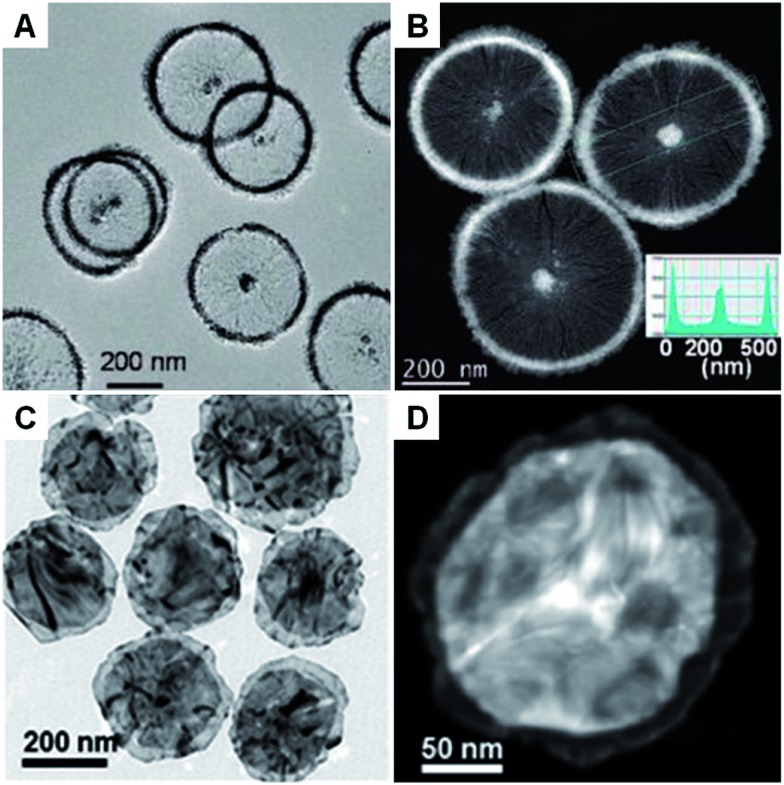
(A) Bright-field TEM and (B) HAADF-STEM images of Pt nanowheels. Inset in (B): Pt density profile across the selected area of a Pt nanowheel shown in (B).^51^ Copyright 2008, American Chemical Society. (C) Bright-field TEM image of Au–Pd nanowheels.^52^ (D) HAADF-STEM image of a typical Au–Pd nanowheel.^52^ Copyright 2013, John Wiley & Sons, Inc.

An ionic surfactant can form micelles when its concentration exceeds the critical micelle concentration (CMC) in aqueous or water–oil mixed solution.^78^ For instance, disc-like micelles called bicelles can be assembled from a mixture of CTAB and sodium perfluorooctanoate (FC7) at a molar ratio of 50 : 50 in water.^51^ With the assistance of this bicelle structure, Pt nanowheels with a diameter of ∼500 nm were successfully obtained *via* the reduction of K_2_PtCl_4_ with ascorbic acid (AA) (Fig. 1A and B).^51^ A following study showed that rectangular dendritic Pt nanosheets with thick centers could be synthesized when the concentration of CTAB/FC7 was increased, which was attributed to the change in surfactant mesophase.^74^ Recently, bimetallic Au–Pd nanowheels, with a yield up to 94%, were prepared *via* the coreduction of Na_2_PdCl_4_ and HAuCl_4_ with AA in the presence of OTAC and HNO_3_ (Fig. 1C and D).^52^ The thickness and average diameter of the Au–Pd nanowheels are ∼6 nm and 290 nm, respectively. It was found that OTAC plays an essential role in the synthesis process because only large aggregated particles were obtained in the absence of OTAC.^52^ Moreover, Cu nanodisks and Ag nanoplates have also been synthesized by using the ionic surfactant-assisted synthesis method.^71–73,75–77^

In addition to the ionic surfactants, polymer surfactants, such as PVP, PVA and P123, have been used as stabilizers in the colloidal synthesis of metal nanoplates and nanosheets.^80,84,92^ As a typical example, Xia *et al.* found that PVP can not only serve as a surfactant but also be used as a reductant to reduce metal salts by its hydroxyl (–OH) end groups.^84^ As a result, thin triangular or hexagonal nanoplates of noble metals including Ag, Pd, Au and Pt were synthesized by using PVP.^84^ Note that the stable metal nanoplates with narrow size distribution and regular shape can be obtained by using PVP as the surfactant, because PVP can selectively bind on particular crystal facets, resulting in the controlled synthesis of anisotropic metal nanostructures. PVA has many –OH groups that can promote the nucleation of metal nanostructures and thus has been used as both a reducing agent and a stabilizer for the synthesis of triangular, pentagonal and hexagonal Au nanoplates, which were prepared in a solid thin film by spin-coating an aqueous solution of HAuCl_4_ and PVA (MW ∼20 000) onto quartz or glass substrates or those pre-coated with polystyrene, and then heated at 100–170 °C for 5–60 min.^87^ Moreover, the block copolymer P123, which can form micelles by simply adjusting the polarity of solvents, has also been used for the synthesis of large triangular and hexagonal Au nanosheets with thickness and width of ∼100 nm and ∼10 μm, respectively.^47^ Recently, a very detailed study of the polymer-assisted synthesis of Ag nanoplates suggests that the physical properties of polymers including length, amphiphilic structure, charge and the types of polymer chains, have significantly affected the synthesis of metal nanostructures.^93^

Nonionic surfactants, such as OA and OAM, have also been used for the synthesis of metal nanoplates and nanosheets in the oil phase at a relatively high temperature. In 2001, Alivisatos *et al.* firstly reported the synthesis of hcp Co nanodisks with a thickness of ∼4 nm from Co_2_(CO)_8_ with a combination of OA and TOPO in *o*-dichlorobenzene at about 200 °C.^32^ They found that the growth rate of different crystal facets can be regulated by changing the composition of the OA and TOPO mixed surfactant. The size of the Co nanodisks is directly proportional to the TOPO concentration if the OA concentration is kept constant. The coexistence of OA and TOPO is critical to guarantee the narrow size distribution and uniform shape of Co nanodisks. In their following study, in addition to OA and TOPO, a series of amines was systematically investigated in order to achieve better control over the size and shape of Co nanodisks and further improve the synthesis yield.^88^ Interestingly, they found that linear amines with longer carbon chain lengths favored the formation and the yield of disks, while the addition of ternary amines completely prohibited the formation of Co disks. Based on these observations, they proposed that the amino (–NH_2_) functional group promotes the formation of Co nanodisks *via* selective adsorption to a particular crystal facet. Another study revealed that OAM can react with an equivalent amount of [Rh(CO)_2_Cl]_2_ to form Rh(CO)_2_Cl(OAM) complexes, which can assemble into molecular wires *via* metallophilic Rh(i)–Rh(i) interactions.^89^ After heating the resulting Rh(CO)_2_Cl(OAM) complex solution at 50 °C for 10 days without stirring, ultrathin Rh nanoplates with a thickness of ∼1.3 nm were obtained by slowly reducing the Rh(i) with OAM. Similarly, the yield of Rh nanoplates was also significantly reduced when the length of the alkyl chains of linear amines was reduced from C_18_ to C_4_, indicating that the van der Waals interactions between the coordinated linear amines is essential to the synthesis of metal nanoplates. Moreover, Au, Ag and Ni–Cu alloy nanoplates and nanosheets have also been synthesized with nonionic surfactants.^90,91,94^

### 2.2. 2D template-assisted synthesis

In the presence of 2D templates, such as lamellar hydrogels,^33,41^ graphene oxide,^28^ graphite^55,95–97^ and lyotropic liquid crystals,^98,99^ metal nanocrystals can be directed or confined to grow into nanoplates and nanosheets under appropriate conditions. For example, an iridescent hexadecylglyceryl maleate (HGM) hydrogel has been used to synthesize single-crystalline ultrathin Au nanosheets with large areas over 100 μm^2^.^41^ It was found that the HGM hydrogel consisted of a periodically regular sandwich structure of lamellar bilayer membranes and 100 nm-thick water layers, which could function as a 2D template to guide the growth of ultrathin Au nanosheets from HAuCl_4_. Note that the surface of these large Au nanosheets is atomically smooth. Another work by the same group demonstrated that the thickness of Au nanosheets can be well-tuned from 5 nm to 40 nm by increasing the concentration of Au precursor (*i.e.* HAuCl_4_) in an iridescent solution of dodecylglyceryl itaconate (DGI).^33^ Similarly, DGI can also form lamellar bilayer membranes, resulting in a periodically ordered structure with water layers between the membrane layers.

**Fig. 2:**
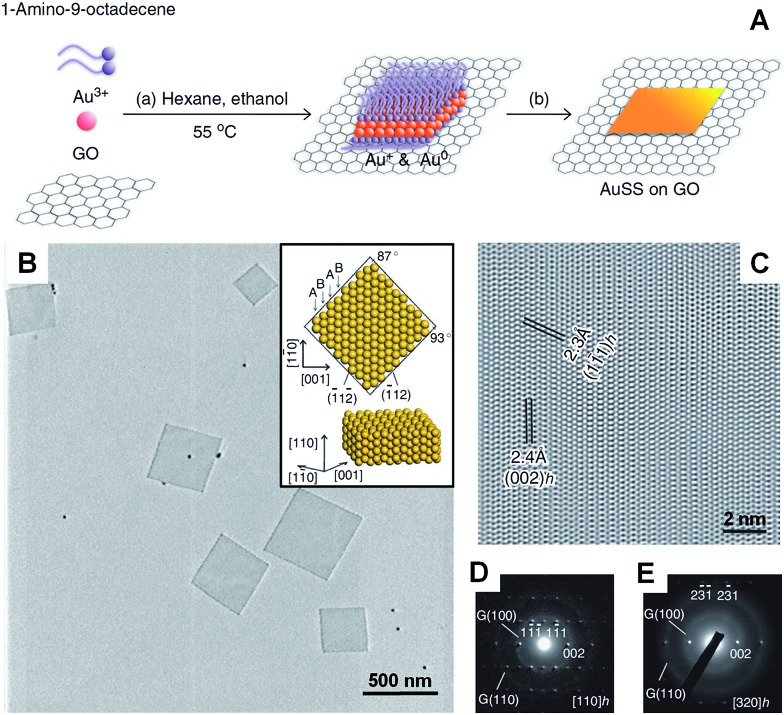
(A) Schematic illustration of the graphene oxide sheet-assisted synthesis of hcp AuSSs.^28^ (B) Bright-field TEM image of the AuSSs on graphene oxide. Inset: crystallographic models of an AuSS shown in (B).^28^ (C) The HRTEM image of an AuSS.^28^ (D and E) SAED patterns of an AuSS taken along the [110]_*h*_ and [320]_*h*_ zone axes, respectively. Note that the two weak diffraction rings are derived from the underlying graphene oxide sheet.^28^ Copyright 2011, Nature Publishing Group.

Recently, our group demonstrated the graphene oxide-templated synthesis of ultrathin AuSSs by slowly reducing HAuCl_4_ with OAM (Fig. 2A and B).^28^ The resultant AuSSs have a thickness of ∼2.4 nm and edge length of 200–500 nm. Surprisingly, it was found that the AuSSs crystallize in an uncommon hcp structure rather than the conventional fcc structure (Fig. 2C and D). This is the first synthesis of a purely hcp Au structure that is stable under ambient conditions *via* a solution-based procedure.

Graphite, as a layer-structured material, has also been used as a template to synthesize Pd, Pt and Ag nanoplates.^55,95–97^ Graphite can be intercalated with metal chlorides (*e.g.* PdCl_2_ and PtCl_4_) between two adjacent graphene layers to form graphite intercalation compounds (GICs).^95^ Typically, Pd and Pt nanoplates can be obtained by reducing PdCl_2_-GIC and PtCl_4_-GIC precursors with hydrogen at high temperature, respectively.^95,96^ A recent study showed that large single crystal triangular and hexagonal Ag nanoplates can be prepared by mildly annealing the Ag nanoparticles that self-organized on highly oriented pyrolytic graphite (HOPG).^55^ It was found that the Ag nanoplates are epitaxially oriented on HOPG, on which Ag atoms diffuse much faster than those on amorphous carbon. Therefore, only small Ag nanoplates were obtained by replacing HOPG with amorphous carbon. The main disadvantage of the solid 2D-templated synthesis is the need to separate the as-formed metal nanoplates or nanosheets from the templates, which may hinder the studies of their properties and further applications.

Recently, lyotropic liquid crystals were employed in the synthesis of Au nanoplates and Pt nanosheets.^98,99^ Moreover, Au nanoplates could be prepared from HAuCl_4_ in the presence of lecithin lamellar liquid crystals,^99^ which can be formed in its high concentration aqueous solution at room temperature. It was suggested that the formation of Au nanoplates is due to the confinement of the regular lamellar liquid crystal structure.^99^ A subsequent study showed the synthesis of Pt nanosheets from Na_2_PtCl_6_ with lyotropic liquid crystals of polyoxyethylene (20) sorbitan monooleate (Tween 80).^98^ As the Tween 80 molecules can form a hexagonal lyotropic liquid crystal phase, the as-prepared Pt nanosheets were rich in hexagonal-shaped nanoholes.

### 2.3. Seeded growth

Compared with the other synthetic methods, the seeded growth of metal nanoplates and nanosheets usually consists of two steps: seed preparation and subsequent growth. The seeded growth usually gives more narrow size and shape distributions and higher yield over those seen with other methods.^73,100^ In 2002, the large-scale preparation of truncated Ag nanoprisms with a thickness of 24 nm was realized by reducing AgNO_3_ with AA in the presence of Ag seeds.^73^ Importantly, the yield of Ag nanoprisms finally reached about 78%. Another study further improved the yield of Ag nanoprisms to as high as 95%, in which the use of poly(sodium styrenesulphonate) was also quite important as it could strongly affect the defect structure of the seeds and thus boost the growth of seeds into nanoprisms.^100^ Indeed, the as-prepared Ag nanoprisms were rich in intrinsic stacking faults along the fcc 111 direction and thus short-range hcp structures were observed. Recently, Xia *et al.* found that the growth mode (lateral growth or vertical growth) of Ag on Ag nanoplate seeds could be controlled by a selective capping agent and thus realized the edge length and thickness control of Ag nanoplates.^101^ As a result, large Ag nanosheets with size of ∼5 μm and thick Ag nanoplates with a thickness of ∼200 nm were obtained by using sodium citrate and PVP as the capping agents, respectively.

**Fig. 3:**
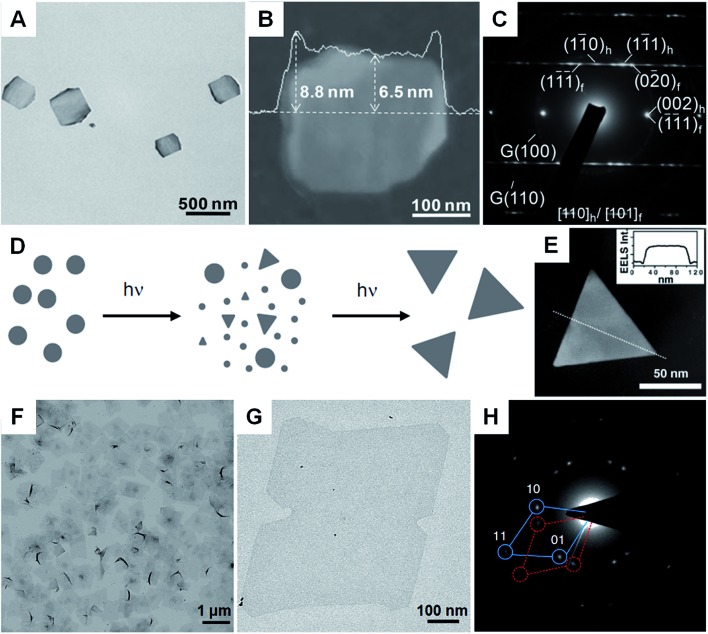
(A) Bright-field TEM image of hcp/fcc mixed Au square plates.^56^ (B) AFM image and the corresponding height profile of an Au square plate.^56^ (C) SAED pattern of an Au square plate taken along the [110]_*h*_/[101]_*f*_ axes.^56^ Copyright 2011, John Wiley & Sons, Inc. (D) Schematic illustration of the photochemical synthesis of Ag nanoprisms.^31^ (E) EELS mapping and the corresponding (inset) EELS intensity profile of a typical Ag nanoprism, showing its flat-top morphology.^31^ Copyright 2001, American Association for the Advancement of Science. (F and G) Bright-field TEM images of Rh nanosheets.^27^ (H) SAED pattern of Rh nanosheets.^27^ Copyright 2014, Nature Publishing Group.

Besides Ag, Au nanoplates have also been successfully synthesized by using the seeded growth method. Mirkin *et al.* reported the synthesis of Au nanoprisms with uniform size and thickness *via* a subsequent three-step growth of Au on the preformed Au seed nanoparticles.^39^ In a following study, they found that the edge length of Au nanoprisms can be modulated between 100 nm and 300 nm by reducing the growth rate of Au onto the Au nanoprism seeds, while the thickness and crystallinity of the initial Au nanoprisms were kept same.^102^ A recent study of seeded growth of Au nanoplates showed that control of edge length and thickness, and simultaneous improvement of the uniformity and yield can be achieved by finely tuning the experimental conditions, such as the time interval between multiple steps of growth and the concentration of CTAB in the solution.^103^ In addition to the aforementioned control of size, shape and yield by seeded growth, our group recently discovered that there is a crystal structure change in the synthesis of square-like Au plates from ultrathin AuSSs (Fig. 3A–C).^56^ Interestingly, the hcp structure of the initial AuSSs transformed to the hcp/fcc mixed structure in the final Au square-like plates, which was most probably due to the thickness increase during the successive growth of Au on the hcp AuSSs.

As is already known, seeded growth is widely applied for the synthesis of bimetallic nanoplates with a core–shell structure.^57,58^ Usually, the reduction potential of the core metal (the seed) should be higher than that of the shell metal in order to avoid the Galvanic reaction happening between them. Based on this consideration, bimetallic core–shell Au@Ag nanoprisms, Au@Cu nanoplates, Au@Ni nanoprisms, Pd@Ag nanoplates, Au@Ag hexagonal nanoplates and Au@Ag nanoplates have been prepared by using Au nanoprisms, Au nanoplates, Au nanoplates, Pd nanosheets, Au nanodisks and Au nanoframes as seeds, respectively.^58,104–108^ Surprisingly, recent studies showed that core–shell bimetallic nanoplates can also be obtained by seeded growth even if the reduction potential of the core metal is smaller than that of the shell metal.^57,109^ It was found that the Galvanic reaction between the core and shell metals can be effectively minimized with the addition of protective capping agents for the core metal and/or simultaneously introducing complexing ligands for the shell metal precursor to decrease its reduction potential. As a result, bimetallic core–shell Pd@Pt and Ag@Au triangular or hexagonal nanoplates were successfully obtained.^57,109^ Recently, Xue *et al.* demonstrated a capping agent-free seeded growth of core–shell Ag@Au nanoprisms, in which the epitaxial growth of Au onto the original Ag nanoprisms, with a thickness of 6 nm, was obtained by slowly and simultaneously introducing HAuCl_4_ and hydroxylamine into the Ag nanoprism solution to avoid the Galvanic reaction.^110^

### 2.4. Photochemical synthesis

Photochemical synthesis is particularly effective for the large-quantity synthesis of Ag nanoplates.^31,111–114^ Although a colloidal suspension of Ag nanoparticles is needed, the photochemical synthesis distinguishes itself from the commonly used seeded growth by the fact that the shape transformation and successive growth from small nanoparticles to large nanoplates are driven by light. Mirkin *et al.* first reported the light-driven synthesis of Ag nanoprisms from spherical Ag particles (∼8 nm in diameter) in the presence of low-concentration trisodium citrate as a reductant and capping agent (Fig. 3D and E).^31^ The size of the obtained Ag nanoprisms can be manipulated by changing the excitation wavelength of the light source.^30^ Importantly, relatively monodisperse Ag nanoprisms were obtained with a dual-beam irradiation of the initial Ag nanoparticles.^30^ Moreover, the size of relatively monodispersed Ag nanoprisms could be gradually adjusted from 38 nm to 120 nm by tuning the primary excitation wavelength from 450 nm to 750 nm. However, illumination for too long a time will induce the shape change in the formed Ag nanostructures from nanoprisms to nanodisks *via* a photoablation process.^115^ Detailed mechanistic studies revealed that the presence of trisodium citrate and O_2_ is essential to the photochemical synthesis of Ag nanoprisms.^116,117^ Moreover, the pH of the growth solution and structural defects in the Ag seed nanoparticles can also affect the light-driven synthesis of Ag nanoprisms.^118,119^ Another study showed that the photo-driven citrate-directed synthesis of Ag nanoprisms from Ag seed nanoparticles proceeded through a coalescence process.^115^ Recently, Hisanori *et al.* found that hexagonal Ag nanoprisms with different sizes could be synthesized *via* directly illuminating an AgNO_3_ solution under monochromatic visible light with different wavelengths.^120^

Besides Ag, nanoplates of bimetallic Au@Ag and Au can also be obtained by using photochemical synthesis.^59,121^ For instance, bimetallic core–shell Au@Ag nanoprisms were synthesized by the light-driven growth of Ag on Au seed nanocrystals.^59^ It was identified that the successive deposition of Ag on Au seed nanocrystals was promoted by activation of the plasmonic Au core by surface plasmon resonance excitation. Meanwhile, the thickness of bimetallic Au@Ag nanoprisms could be tuned by changing the size of the Au seed nanocrystals. Recently, our group showed the photochemical synthesis of six-star Au nanoplates from Au seed nanoparticles in the presence of TiO_2_ sol.^121^ The six-star Au nanoplates exhibit a thickness of 60–80 nm and a size of 250–350 nm. The colloidal TiO_2_ sol was used as a photocatalyst to reduce the Au precursor and stabilize the as-formed six-star Au nanoplates.

### 2.5. Hydrothermal and solvothermal methods

Hydrothermal and solvothermal methods have been extensively used in the synthesis of various kinds of nanomaterials due to their facile operation and simple setup.^122^ However, compared to the other methods, these methods are relatively energy-consuming as high temperature is usually required. Typically, the synthesis is conducted in a sealed autoclave at elevated temperature and pressure. Under such experimental conditions, water and non-aqueous solvents display variation in properties such as viscosity and dissociation constant. Recently, hydrothermal methods have been successfully employed in the synthesis of metal nanoplates.^61,123^ For example, Yan *et al.* prepared ultrathin triangular Ru nanoplates with a thickness of ∼3.0 nm by reducing RuCl_3_·*x*H_2_O with HCHO (40 wt%) in the presence of PVP at 160 °C for 4 h.^61^ By simply increasing the concentration of RuCl_3_·*x*H_2_O and PVP, irregular-shaped ultrathin Ru nanoplates with a thickness of ∼1.5 nm were obtained. Another study showed the hydrothermal synthesis of triangular Ag nanoplates with straight and curved edges from AgNO_3_ in the presence of PVP at 160 °C for 4 or 6 h.^123^

A solvothermal method that uses non-aqueous solvents has been used for the synthesis of metal nanoplates and nanosheets.^27,34,124,125^ Notably, Li *et al.* reported the solvothermal synthesis of single-atom layer Rh nanosheets with edge lengths of 500–600 nm from Rh(acac)_3_ (acac = acetylacetonate) in a mixture of PVP, benzyl alcohol and formaldehyde at 180 °C for 8 h (Fig. 3F–H).^27^ Interestingly, the as-prepared Rh nanosheet with a hexagonal structure is different from the cubic structure commonly observed in Rh nanocrystals. Wang *et al.* demonstrated the solvothermal synthesis of ultrathin bimetallic Pt–Cu alloy nanosheets with thicknesses ranging from 1.32 to 0.88 nm *via* a two-step solvothermal method, *i.e.* the preparation of a gel-like material that was then used in the subsequent growth process of Pt–Cu nanosheets.^34^ Importantly, the size of the formed Pt–Cu nanosheets could be tuned from 8 to 50 nm by simply increasing the concentration of KI in the growth solution. In addition, metal nanoplates such as Ag nanoprisms and bimetallic Bi–Rh nanoplates have also been successfully prepared *via* the solvothermal synthesis method.^125–127^

### 2.6. Carbon monoxide and halide ion confined growth

**Fig. 4:**
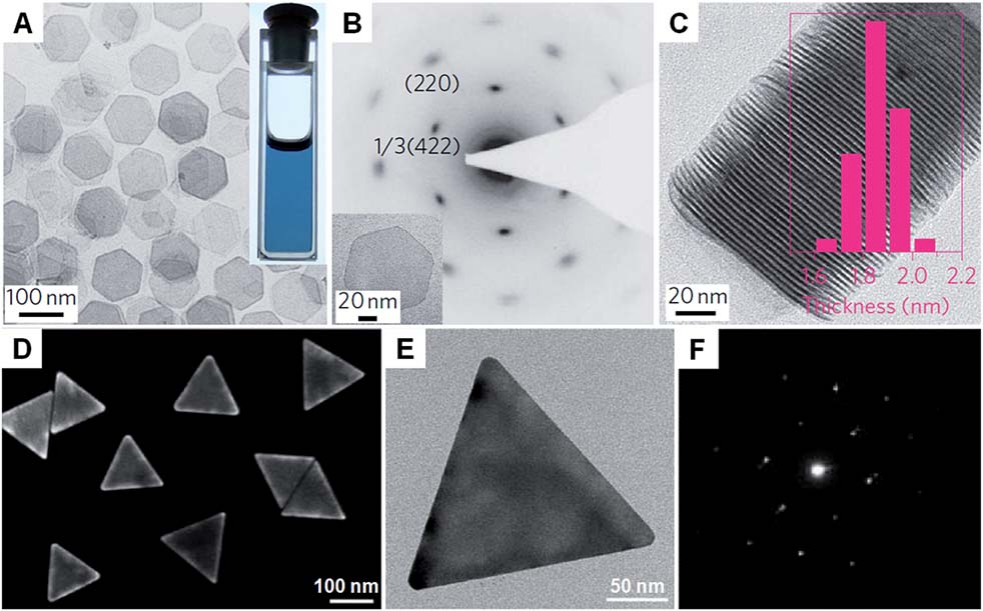
(A) Bright-field TEM image of free-standing Pd nanosheets. Inset: a photograph of the Pd nanosheet solution.^29^ (B) SAED pattern of a Pd nanosheet (inset) taken along the [111] zone axis.^29^ (C) TEM image and the corresponding thickness analysis (inset) of the assembly of Pd nanosheets.^29^ Copyright 2011, Nature Publishing Group. (D) SEM image of Au nanoprisms.^62^ (E) Bright-field TEM image and (F) the corresponding SAED pattern of a typical Au nanoprism.^62^ Copyright 2013, American Chemical Society.

Carbon monoxide (CO) is known for its poisoning effect in metal catalysis as it can strongly adsorb on to the surface of metals (*e.g.* Pd).^128^ Surface studies have revealed the strong adsorption of CO on low-index facets of Pd crystals, such as fcc (111) planes.^129–131^ Meanwhile, CO is an effective reducing agent of various Pd salts, such as Pd(acetate)_2_ and Pd(acac)_2_.^29,132^ Therefore, it has been successfully used for the synthesis of many kinds of Pd nanocrystals.^133^ Because of the extremely strong interaction between Pd and CO molecules, ultrathin Pd nanosheets (1–2 nm in thickness) can be obtained in the presence of CO. In 2009, ultrathin hexagonal Pd nanosheets with a thickness of ∼2 nm were first prepared with ternary emulsions in the presence of CO.^132^ However, the size and shape of the formed Pd nanosheets were not uniform. In 2011, Zheng *et al.* realized the synthesis of freestanding Pd nanosheets with regular hexagonal shape and narrow size distribution *via* reducing Pd(acac)_2_ with CO in a mixture of PVP, DMF (or benzyl alcohol), water and a halide salt at 100 °C for 3 h under stirring (Fig. 4A–C).^29^ Importantly, the edge length of the obtained Pd nanosheets can be tuned from 20 nm to 160 nm. The use of CO is essential to the synthesis of Pd nanosheets since only twinned Pd nanoparticles are obtained in the absence of CO. As expected, CO stripping voltammetry confirmed that the CO molecules bound tightly on the (111) surfaces of the Pd nanosheets and thus confined the Pd nanocrystals to grow into a sheet structure. Moreover, by changing the solvent from DMF or benzyl alcohol to dimethylactamide or *N*,*N*-dimethylpropionamide, ultrasmall Pd nanosheets with a size of ∼4.4 nm were obtained.^134^ Interestingly, mesocrystalline Pd nanocorollas made of Pd nanosheets were obtained with the addition of a small amount of FeCl_3_.^135^ Recently, Zheng *et al.* found that ultrathin Pd nanosheets can be directly prepared by simply mixing a Pd carbonyl complex ([Pd_2_(μ-CO)_2_Cl_4_]^2–^) with water in the absence of any surfactant, in which CO serves as the reductant of Pd(i).^38^ However, it should be noted that the synthesis of ultrathin metal nanosheets assisted with CO has been limited to Pd so far.

Recently, halide ions have been widely employed for the synthesis of metal nanoplates as they can selectively adsorb on to the metal surface.^62,136^ For example, the iodide ion plays an important role in the synthesis of Au nanoprisms.^44^ Even a small amount of iodide ions (∼20 μM) can effectively promote the growth of Au nanoprisms with a high yield. It was confirmed that iodide ions can strongly and selectively bind to the Au (111) planes and thus favor the formation of (111)-oriented Au nanoprisms.^137^ Another study revealed that both bromide and iodide ions are necessary to prepare high-quality Au nanoprisms (Fig. 4D–F).^62^ However, chloride ions are not effective and a high concentration of chloride ions will prohibit the formation of Au nanoprisms. Recently, intermetallic Pt–Bi nanoplatelets with a thickness of ∼6 nm were synthesized from a mixture of Pt(acac)_2_, Bi(neodecanoate)_3_, OAM and NH_4_Br at 90 °C for 30 min.^138^ The size of the Pt–Bi nanoplatelets can be tuned from 20 nm to 80 nm by changing the reaction time from 1 min to 10 min. It was found that the bromide ions are critical in the synthesis of Pt–Bi nanoplatelets as halide ions can selectively adsorb on the crystal surface. Indeed, Pt–Bi nanoplatelets can also be obtained by replacing NH_4_Br with NH_4_F, NH_4_Cl, or NH_4_I.

### 2.7. Self-assembly of nanoparticles

**Fig. 5:**
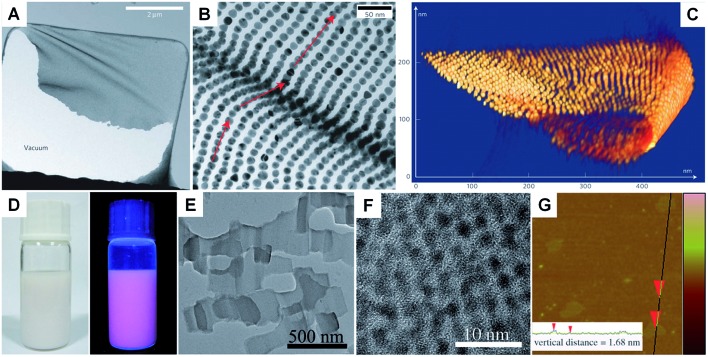
(A) Low-magnification TEM image of a crumpled Au nanoparticle superlattice sheet partially attached to a hole in the carbon substrate.^67^ (B) High-magnification TEM image of a wrinkle shown in (A), showing that the Au nanoparticles remain aligned despite the fact that the nanoparticle superlattice sheet is bent.^67^ (C) 3D STEM tomography reconstruction of a typical folded Au nanoparticle superlattice sheet.^67^ Copyright 2009, Nature Publishing Group. (D) Photograph (left) and the corresponding photoluminescence (right) of the self-assembled Au_15_ cluster nanosheet dispersion.^66^ (E) Low-magnification TEM image of the Au_15_ cluster nanosheets.^66^ (F) HRTEM image of a typical Au_15_ cluster nanosheet.^66^ (G) AFM image and the corresponding height analysis (inset) of the Au_15_ cluster nanosheets.^66^ Copyright 2013, John Wiley & Sons, Inc.

Unlike the other synthetic methods, self-assembly uses metal nanoparticles as a basic building block.^139–141^ Usually, self-assembly of metal nanoparticles can induce the formation of metal nanosheets with a much larger size at the microscale and even larger compared to those obtained by other methods.^67^ Moreover, this method induces the formation of metal nanosheets with a superlattice structure rather than the single crystal structure prepared with other methods. In general, self-assembly-mediated formation of metal nanosheets can be realized by solvent evaporation at an interface (*e.g.* air–liquid and liquid–liquid interfaces) or using molecular linkers (*e.g.* DNA).^66,67,139–142^ For example, Luo *et al.* reported the fabrication of free-standing monolayered Au nanoparticle superlattice sheets with 5′-thiolated single-stranded DNA (ssDNA) capped Au nanoparticles (13 nm in diameter) (Fig. 5A–C).^67^ The Au nanoparticle superlattice sheets with highly ordered hexagonal packing were assembled *via* microhole-confined self-assembly, which was carried out by drying a droplet of an Au nanoparticle solution on to a holey substrate and thus diminishing the nanoparticle-to-substrate interaction. Importantly, the edge-to-edge inter-particle spacing of the monolayered Au nanoparticle superlattice sheets can be modulated up to 20 nm, which is much larger than that formed with the commonly used alkyl thiol molecules. Recently, Zhang *et al.* demonstrated the preparation of ultrathin monolayered Au_15_ nanocluster sheets with a length of 200–1000 nm and a width of ∼300 nm *via* self-assembly at the liquid–liquid interface under vacuum (Fig. 5D–G).^66^ Interestingly, Au_15_ nanocluster sheets with relatively uniform size and regular shape were obtained when N_2_ was introduced to lower the solvent evaporation rate. Besides, it was found that the formation of Au_15_ nanocluster sheets is favored at a high liquid paraffin/dibenzyl ether ratio and high concentration of Au_15_ nanoclusters. Importantly, the low ratio of liquid paraffin/dibenzyl ether and low concentration of Au_15_ nanoclusters can induce a shape transformation from sheets to bipyramids, nanospheres, or irregular aggregates.

## 3. Properties

### 3.1. Crystal structure

Metal nanoplates and nanosheets are mostly made of close-packed metal atoms connected together by metallic bonds.^37^ Taking Au as an example, Au nanoplates and nanosheets have two polytypes, fcc and hcp, showing characteristic stacking sequences of “ABCABC…” and “ABAB…” along the close-packed direction of [111]_*f*_ and [001]_*h*_, respectively. As the bulk Au crystal has an fcc phase, the structure of Au nanoplates and nanosheets, such as triangular and hexagonal Au nanoplates and nanosheets, is mainly dominated by the fcc type.^33,39,42^ The fcc Au nanoplates and nanosheets usually demonstrate an orientation of (111)_*f*_ and are mainly enclosed by the low-index facet of {111}_*f*_, which has the lowest surface energy compared to the {100}_*f*_ and {101}_*f*_ planes.^37^ Interestingly, an ultrathin Au nanosheet has been observed to crystallize in the hcp lattice type, such as hcp AuSS with a thickness of ∼2.4 nm, which exhibits the orientation of (110)_*h*_ and owns a {110}_*h*_ basal plane.^28^ Differently, the hcp nanosheets of both Ru and Rh show orientation of (001)_*h*_ and are mainly enclosed by the {001}_*h*_ facets.^27,61^

### 3.2. Optical properties

**Fig. 6:**
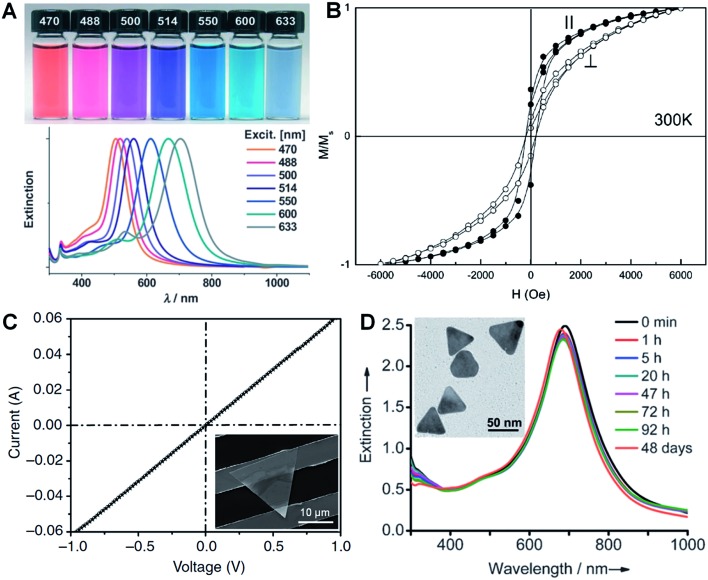
(A) Photographs (top) and the corresponding surface plasmon resonance spectra (bottom) of Ag nanoprisms prepared with different excitation wavelengths.^118^ Copyright 2007, John Wiley & Sons, Inc. (B) Magnetic hysteresis loops of Co nanoplatelets with the magnetic field applied parallel (‖) and perpendicular (⊥) to the nanoplatelets at 300 K. *M*/*M*_s_ represents the magnetization (*M*) normalized to the saturated magnetization (*M*_s_).^148^ Copyright 2007, American Chemical Society. (C) Current–voltage (*I*–*V*) curve of an Au membrane (∼5 nm in thickness) deposited on two Au electrodes with a channel length of 10 μm. Inset: optical image of the corresponding device measured in (C).^41^ Copyright 2014, Nature Publishing Group. (D) Stability evaluation of Ag@Au nanoplates in a H_2_O_2_ solution (2.1%) by monitoring the UV-vis-NIR spectrophotometry at different times. Inset: a TEM image of the Ag@Au nanoplates after treatment in H_2_O_2_ solution for 2 h.^109^ Copyright 2012, John Wiley & Sons, Inc.

LSPR is a collective electronic oscillation of the surface-conduction electrons of metal nanostructures excited by electromagnetic radiation.^143^ The LSPR of a metal nanostructure is determined by its size, shape, dimensionality and composition.^29,104,144^ During the last two decades, plasmonic Au and Ag metal nanomaterials were extensively studied due to their distinct dielectric properties in the vis-NIR spectral region and low intrinsic loss.^143^ In particular, nanoplates and nanosheets of Au^39,43,94^ and Ag^30,31,100,145^ (*e.g.* nanoprisms) have received great attention because of their broad spectral absorption with an especially strong plasmon absorption in the NIR region compared to the nanoparticles. Taking Ag as an example, Mirkin *et al.* first revealed that the Ag nanoprisms showed two distinct quadrupole plasmon resonance peaks besides the two dipole plasmon resonance peaks.^31^ Meanwhile, the Ag nanoprisms exhibited a Rayleigh scattering in the red color region, which has not been observed from either Au or Ag spherical nanoparticles.^31^ Importantly, the plasmon resonance peaks of the Ag nanoprisms can be efficiently tuned over a wide range by simply changing their size (Fig. 6A).^30,100,118^ In addition, it was revealed that the environmental refractive index and Au coating of the Ag nanoprisms can also significantly affect the position of their plasmon resonance peaks.^110,146^ Recently, broad spectral and strong NIR plasmon absorptions have been observed in a range of nanoplates and nanosheets of other metals, such as Al nanodisks and nanoprisms,^70,147^ Cu nanoplates and nanoplatelets,^86,148^ triangular and hexagonal Ni nanosheets,^149^ and Pd nanoplates and hexagonal nanosheets.^29,40,84,132^ Notably, the ultrathin Pd hexagonal nanosheet demonstrated a relatively high extinction coefficient in the NIR region, which is comparable to that of the Au plasmonic nanostructure.^29^ Moreover, the position of the NIR plasmon absorption peak of the ultrathin Pd nanosheet can be easily tuned by either changing its edge length or coating a thin layer of Ag on it.^29,104^

### 3.3. Magnetic properties

Magnetic phenomena are commonly observed in Fe-, Co- or Ni-based metal materials. Isotropic Fe-, Co- or Ni-based metal nanoparticles are usually superparamagnetic at ambient conditions, which hinders their application in magnetic recording.^148^ Since the magnetic anisotropy can be modulated by controlling the shape of nanocrystals, Fe-, Co- or Ni-based metal nanoplates and nanosheets are expected to give enhanced magnetic properties.^91,148–151^ Li *et al.* found that both the coercivity forces and blocking temperatures of Co and Ni nanoplatelets are much larger compared to their bulks and isotropic nanoparticles.^148^ Notably, the Co nanoplatelets showed coercivities of 218 Oe and 176 Oe with magnetic fields applied parallel and perpendicular to the nanoplatelets at 300 K, respectively (Fig. 6B). A recent study showed an increased coercivity of a Co thin film, *i.e.* 321.7 Oe obtained in a parallel magnetic field and 220.5 Oe obtained in a perpendicular magnetic field, composed of Co nanosheets with a larger size compared to the aforementioned Co nanoplatelets.^150^ Meanwhile, it was found that triangular Ni–Cu alloy nanoplates exhibited a higher block temperature than those of their hexagonal counterparts, which is attributed to the higher degree of anisotropy of the triangular plate compared to the hexagonal one.^91^ Moreover, another study revealed that the larger the edge length of Ni nanosheets, the higher the block temperature and magnetization.^149^

### 3.4. Electrical properties

In view of their metallic conductivity and flexibility, Au nanosheets exhibited great potential for various electronic applications.^152^ For instance, highly stretchable Au electrodes with a relatively low electrical resistivity in the order of 10^–6^ Ω m have been prepared with large Au nanosheets.^152^ Moreover, the Au nanosheet electrode showed an excellent electrical stability at 100% strain during the cycling test. Recently, Jin *et al.* studied the electrical properties of an Au membrane with a thickness of ∼5 nm (Fig. 6C).^41^ The Au membrane demonstrated conventional ohmic behavior with an extremely low electrical resistivity, 4.0 × 10^–8^ Ω m, under ambient conditions. Importantly, the electrical resistivity of the Au membrane is very close to that of bulk Au (2.2 × 10^–8^ Ω m) and 1–2 orders of magnitude lower than that of the thermally evaporated Au film, which might be attributed to the single-crystalline nature of the Au membrane. Meanwhile, it also exhibited high transparency of about 80% under light between 450 nm and 700 nm. Therefore, the as-prepared Au membrane is promising for transparent electrodes.

### 3.5. Stability

Due to the anisotropic and ultrathin characteristics of metal nanoplates and nanosheets (*e.g.* sharp corners and curved edges with high surface energy), they are prone to degrade under harsh chemical or thermal circumstances.^28,153–157^ For example, halide ions (*e.g.* Cl^–^, Br^–^ and I^–^) and SCN^–^ ions can sculpt the regular Ag nanoprisms into Ag nanodisks at room temperature.^157^ Ag nanoprisms were found to undergo a shape transition from triangular to disk-like upon modification with thiol-terminated poly(ethylene glycol) (PEG-SH, 0.1 mM).^156^ Moreover, the Ag nanoprisms evolved into irregular Ag nanoparticles after treatment with PEG-SH (1.0 mM) for 20 h under ambient conditions. Meanwhile, Ag nanoprisms coated with SiO_2_ were observed to start degrading when the temperature was above 62 °C.^153^ Further, we found that the ultrathin AuSSs (Fig. 2) are very sensitive to the electron beam.^28^ A very short duration of electron beam illumination (∼5 s at 200 keV) could induce a phase transformation from hcp to fcc with crystal defects, *e.g.* stacking faults and twins, along with a morphology change. Recently, it was found that coating a thin layer of another metal or alloying with another metal to form bimetallic nanostructures can greatly enhance the stability of a particular metal nanoplate or nanosheet.^104,109,158,159^ For example, Yin *et al.* observed that epitaxial growth of a thin layer of Au on the pristine Ag nanoplate can significantly increase its stability towards treatment with the relatively strong oxidant H_2_O_2_ (Fig. 6D).^109^

## 4. Applications

### 4.1. Catalysis

**Fig. 7:**
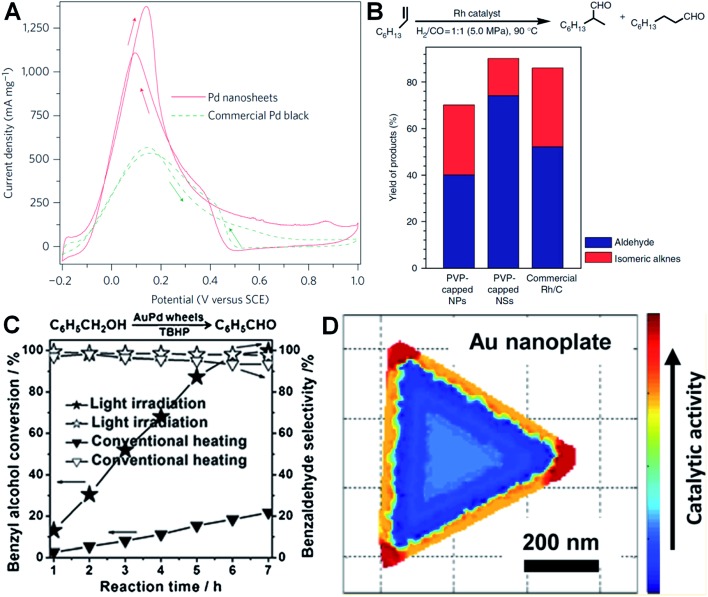
(A) Cyclic voltammetry (CV) curves of Pd nanosheets (edge length: 41 nm) and Pd black (Aldrich, 47 m^2^ g^–1^) collected in an aqueous solution containing H_2_SO_4_ (0.5 M) and HCOOH (0.25 M) at a scan rate of 50 mV s^–1^.^29^ Copyright 2011, Nature Publishing Group. (B) The catalytic ability comparison of PVP-capped Rh nanoparticles (NPs), PVP-capped Rh nanosheets (NSs) and commercial Rh/C in hydroformylation of 1-octene.^27^ Copyright 2014, Nature Publishing Group. (C) The comparison of light irradiation (power density: 0.37 W cm^–2^) and conventional heating (temperature: 50 °C) in the selective oxidation of benzyl alcohol with Au–Pd nanowheels as the catalyst.^52^ Copyright 2013, John Wiley & Sons, Inc. (D) Catalytic activity mapping of an Au nanoplate in the reductive N-deoxygenation of resazurin to resorufin by NH_2_OH, identified by the combination of single-molecule super-resolution fluorescence microscopy and electron microscopy.^163^ Copyright 2013, American Chemical Society.

Metal nanoplates and nanosheets, especially those of noble metals, exhibit relatively large surface areas, thus making them promising candidates in various catalytic applications.^27,29,34,38,91,97,124,160^ Notably, some of the noble metal nanoplates and nanosheets demonstrate better catalytic performances over the commercially available catalysts in electrocatalysis.^29,34,38,138^ For instance, Zheng *et al.* reported that Pd nanosheets showed much a larger electrochemically active surface area (ECSA) (67 m^2^ g^–1^) for the oxidation of formic acid compared to that of the commercial Pd black (47 m^2^ g^–1^).^29^ Importantly, the maximum current density of Pd nanosheets was observed to be 1380 mA mg^–1^ at 0.14 V, 2.5 times of that of commercial Pd black (Fig. 7A). In order to further improve their catalytic activity, surface-clean Pd nanosheets were prepared since the surface capping agent on Pd nanosheets is not favored in electrocatalysis. It was found that the surface clean Pd nanosheets gave a maximum current density of 1420 mA mg^–1^ at a much more negative potential (0.04 V) compared to that of PVP-capped Pd nanosheets (0.14 V).^38^ Furthermore, the hybridization of noble metals with earth-abundant transition metals to construct bimetallic nanomaterials is one of the effective ways to reduce the used amount of noble metals and further improve the catalytic performance of noble metal nanoplates and nanosheets.^34,138^ For example, Wang *et al.* reported that the Pt–Cu alloy nanosheet exhibited large ECSA (62.8 m^2^ g^–1^) for the oxidation of ethanol, and its maximum current density is 14 and 9 times those of commercial Pt black and Pt/C, respectively.^34^ Recently, it was revealed that the Pt–Bi nanoplatelet displayed excellent catalytic stability and performance for the oxidation of methanol, giving a final current value 6.4 times that of the commercial Pt/C after 4000 s.^138^

Besides electrocatalysis, noble metal nanoplates and nanosheets also exhibited superior catalytic activities in the catalysis of organic reactions over the commercial catalysts.^27,52,61,160^ For example, it was found that thin Rh nanosheets gave a >99.9% conversion of phenol within 4 h at near room temperature (30 °C) under low H_2_ pressure (1.0 MPa), and exhibited an activity that was 4 and 7 times those of commercial Rh/C and PVP-capped Rh nanoparticles, respectively.^27^ Importantly, the as-prepared Rh nanosheets exhibited both superior catalytic activity and selectivity in the hydroformylation of 1-octene compared to the commercial Rh/C under mild reaction conditions (Fig. 7B). Tuning the surface wettability of noble metal nanoplates and nanosheets is an effective strategy to optimize their catalytic performance since it can modulate the interaction between the metal surface and reactants.^160^ Zheng *et al.* used C_2_H_2_ to treat the surface-clean Pd nanosheet and modify its surface to be hydrophobic.^160^ As a result, the C_2_H_2_-treated Pd nanosheet showed an about 10 times enhanced catalytic activity in styrene hydrogenation compared to the untreated one. In addition, as noble metal nanoplates and nanosheets have very strong LSPR absorption, light irradiation can be used to replace conventional heating in liquid-phase catalysis.^52^ Interestingly, it was observed that the Au–Pd nanowheel exhibited a much higher catalytic activity for the oxidation of benzyl alcohol under light irradiation compared to that obtained with heating (Fig. 7C).^52^

It is very important to identify where the catalysis occurs and which kind of structures are more reactive.^161,162^ Recently, Chen *et al.* developed an effective method for the mapping of the catalytic location and activity in single metal nanostructures.^161,163^ For example, the location of a catalytic reaction on an individual Au nanoplate can be determined with a combination of super-resolution fluorescence microscopy and scanning electron microscopy.^163^ By these means, they revealed the site-specific activities in different areas of an Au nanoplate with an activity order of the corner region > the edge region > the flat surface plane (Fig. 7D).

### 4.2. Surface enhanced Raman scattering

**Fig. 8:**
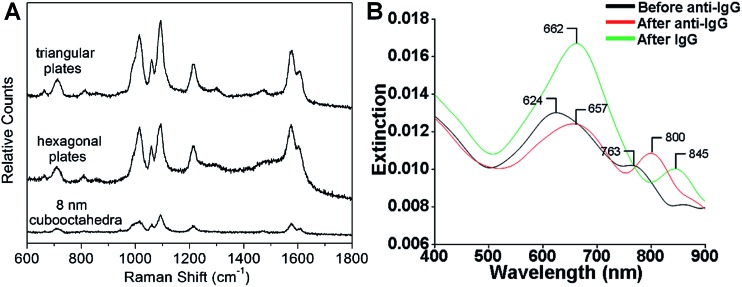
(A) Surface enhanced Raman scattering (SERS) spectra of 4-mercaptopyridine adsorbed on to Pd triangular nanoplates, hexagonal nanoplates and cuboctahedra.^40^ Copyright 2005, American Chemical Society. (B) LSPR spectra of a glass sample coated with Au nanoplates before functionalization with human anti-IgG (black curve), after functionalization with human anti-IgG with 6 mM place-exchanged 11-mercaptoundodecanoic acid (red curve), and after exposure to 10 pg mL^–1^ human IgG (green curve).^169^ Copyright 2010, American Chemical Society.

SERS is one of the most powerful tools for the detection of various molecules due to its high sensitivity and selectivity.^69,164–167^ Noble metal nanomaterials, especially Ag and Au, have been proven to be most effective among all the SERS substrates.^143,165,166^ Anisotropic metal nanoplates and nanosheets are expected to possess remarkable SERS activities because of the presence of sharp corners, which show extremely strong electromagnetic fields and thus produce Raman “hot spots” that are not present in isolated spherical nanoparticles.^46,164,167,168^ For example, Xia *et al.* reported that the SERS signal intensity of 4-mercaptopyridine from triangular and hexagonal Pd nanoplates was 4.3 and 3.4 times that of Pd cuboctahedra (Fig. 8A), which experimentally proved the importance of sharp corners in SERS.^40^ Therefore, introducing more sharp tips in metal nanoplates and nanosheets may further enhance their Raman signals. Recently, Qin *et al.* systematically studied the SERS activities of Ag nanoplates with straight, curved and wavy edges.^123^ Compared to Ag nanoplates with straight and curved edges, those with wavy edges are richest in sharp corners and this can significantly enhance the localized electromagnetic field. As expected, the Ag nanoplates with wavy edges displayed the strongest SERS signal for 1,4-benzenedithiol molecules among the aforementioned three kinds of Ag nanoplates. However, the sharp corners in metal nanoplates and nanosheets are not very stable. In order to overcome this issue, bimetallic Pd@Ag core–shell nanoplates were prepared for SERS.^104^ Intriguingly, the bimetallic Pd@Ag nanoplates showed a relatively stable SERS signal (decreased less than 10%) for 4-pyridinethiol compared to Ag nanoprisms (decreased as much as 60%) even under laser irradiation (808 nm, 2 W) for 40 min.

The Raman “hot spot”, preferred for SERS, can also be obtained *via* the formation of a narrow gap with plasmon coupling between two metal nanoplates.^46,69,164^ It was theoretically suggested that the maximum amplitude of the normalized electric field of an Ag nanoplate dimer with a gap distance of 2 nm is almost 25 and 200 times that of an isolated single Ag nanoplate and nanoparticle, respectively.^164^ In a related work, SERS of three types of Au nanodisk arrays with different diameters and gratings were measured.^69^ Similarly, it was found that Au nanodisk arrays with smaller diameter and grating showed a stronger SERS signal because of the larger electromagnetic coupling between adjacent Au nanodisks. In addition, it was found that the SERS signal on the assembled structures of Ag nanoprisms could be modulated based on the orientation of Ag nanoprisms.^168^

### 4.3. LSPR-based sensing

The LSPR of metal nanoplates and nanosheets is extremely sensitive to the local dielectric constant of the surrounding environment, which makes them promising for refractive index sensing by monitoring the LSPR peak shift.^146,158,169–173^ For example, Mirkin *et al.* found that an Ag nanoprism monolayer on a glass substrate exhibited an obvious color variation by changing its state from wet to dry.^146^ Detailed experiments revealed that the LSPR peak of the Ag nanoprism monolayer showed a large red shift of about 100 nm by increasing the local dielectric constant from 1.003 (air) to 1.509 (pyridine). In addition, a change in the local dielectric environment of metal nanoplates and nanosheets can also be obtained by adsorption of biomolecules on their surface.^68,169,170,173^ Therefore, metal nanoplates and nanosheets can be utilized for detection of biomolecules. For instance, the LSPR peak of Ag nanoplates showed a red shift upon the binding of human anti-IgG to their edges and the shift increased with the amount of anti-IgG on the edges (Fig. 8B).^169^ Importantly, the LSPR peak shift on Au nanoplates was 4–8 times of that of spherical Au nanoparticles under the same conditions. Remarkably, the Au nanoplates functionalized with anti-IgG exhibited a large LSPR peak shift of 45 nm even for IgG (<1 pM) solution with a significantly low concentration of 10 pg mL^–1^ (Fig. 8B).

The LSPR of metal nanoplates and nanosheets is also highly dependent on their shapes.^144^ As the sharp corners and edges of metal nanoplates and nanosheets (*e.g.* Ag nanoprisms) are prone to deform due to high surface energy, they can be used for the efficient detection of chemicals that can induce a morphology change.^174^ Recently, Chen *et al.* developed a facile colorimetric approach for Hg^2+^ ion detection based on Hg^2+^-induced deprotection and shape transformation of thiol-capped Ag nanoprisms in the presence of I^–^ ions.^174^ The LSPR peak of the Ag nanoprisms showed a blue shift upon the addition of Hg^2+^ ions, which induced the shape change from Ag nanoprisms to nanodisks in the presence of I^–^ ions. Importantly, a relatively low detection limit of 3.3 nM was achieved for Hg^2+^ ions. Moreover, it was also observed that the surface-electron charging of metal nanoplates can lead to the LSPR shift.^175^ Therefore, Ag nanoplates have been successfully used for the detection of individual anions (*e.g.* phosphate, halide and thiocyanate ions) and a relatively high sensitivity in the order of 1 × 10^–6^ M was obtained.^175^

### 4.4. NIR photothermal therapy

**Fig. 9:**
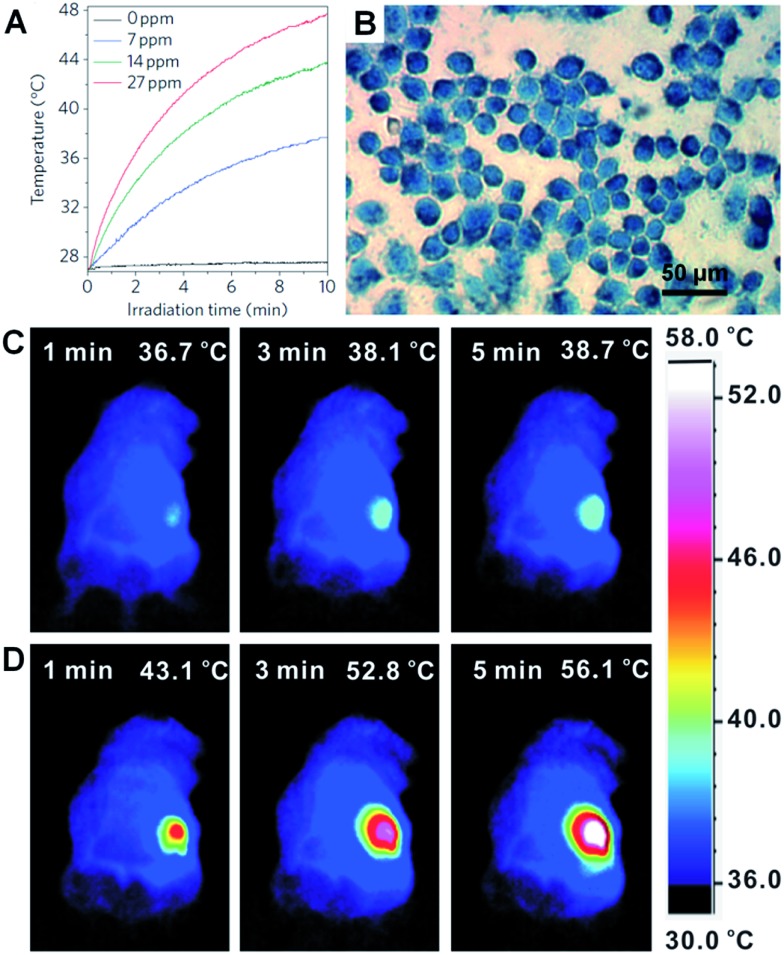
(A) NIR photothermal effect of ultrathin Pd nanosheets with various concentrations in water irradiated by a laser (808 nm, 1 W).^29^ (B) Micrograph of *in vitro* photothermal therapy of liver cancer cells with polyethyleneimine (PEI) modified ultrathin Pd nanosheets under the irradiation of a NIR laser (808 nm, 1.4 W cm^–2^) for 5 min. The dead liver cancer cells are stained with trypan blue.^29^ Copyright 2011, Nature Publishing Group. (C and D) Thermographic images of *in vivo* photothermal therapy of tumor-bearing mice with saline (C) and glutathione-functionalized ultra-small Pd nanosheets (D) under irradiation of a NIR laser (808 nm, 1 W cm^–2^) at different times.^134^ Copyright 2014, John Wiley & Sons, Inc.

Metal nanoplates and nanosheets could be promising candidates for applications in NIR photothermal therapy owning to their strong LSPR absorptions in the NIR region, outstanding photothermal conversion efficiencies, excellent biocompatibilities and photothermal stabilities.^29,45,104,118,134,135,176^ For example, the NIR photothermal effect of ultrathin Pd nanosheets was investigated by monitoring the temperature change of a Pd nanosheet aqueous solution illuminated by a NIR laser.^29^ It was found that the temperature of 1 mL of an aqueous solution of Pd nanosheets with concentration of 27 ppm increased to 48.7 °C from 28.0 °C after 10 min of irradiation with a NIR laser (1 W, 808 nm) (Fig. 9A). In comparison, the temperature of 1 mL of aqueous solution without the addition of Pd nanosheets increased by only 0.5 °C under the same conditions. Importantly, liver cell incubation in the presence of Pd nanosheets showed only a 20% decrease in initial live cell count after 48 h of exposure to a 600 μg mL^–1^ solution of Pd nanosheets, suggesting the excellent biocompatibility of the Pd nanosheets. *In vitro* NIR photothermal therapy based on liver cancer cells revealed that ∼100% of cancer cells were killed under irradiation with a NIR laser (808 nm, 1.4 W cm^–2^) for just 5 min (Fig. 9B). In order to further increase the efficiency of cancer cell therapy, Pd nanosheets were deposited on hollow mesoporous silica spheres (containing drug, *i.e.* doxorubicin) to combine the photothermal therapy and chemotherapy *via* a synergistic effect.^177^ In addition, due to the higher bulk melting point of Pd compared to that of Au and Ag (*T*_mp, Pd_ = 1828 K, *T*_mp, Au_ = 1337 K, and *T*_mp, Ag_ = 1235 K, respectively), the Pd nanosheet was observed to exhibit enhanced photothermal stability compared to Au and Ag nanoprisms under irradiation with a NIR laser (808 nm, 2 W) for 30 min.^29^ Meanwhile, it was reported that the NIR photothermal stability of Pd nanosheets could be further increased by coating with a thin layer of Ag.^104^

However, as the size of the aforementioned Pd nanosheets is relatively large, their efficient renal clearance remains a big problem and thus severely hampers their applications in *in vivo* NIR photothermal therapy.^134^ Usually, the size of nanosheets for *in vivo* experiments should be smaller than 10 nm (the renal filtration limit size) in order to achieve an efficient renal clearance and low biotoxicity. Recently, Zheng *et al.* prepared ultra-small Pd nanosheets with a size of about 4.4 nm for *in vivo* photothermal therapy.^134^ The Balb/c mice bearing 4T1 tumors were randomly divided into two groups that were injected with 200 μL of glutathione-modified ultra-small Pd nanosheets (2 mg mL^–1^) and saline, respectively. The 4T1 tumor was irradiated with a NIR laser (808 nm, 1 W cm^–2^) for 5 min, 24 h post injection. As expected, the surface temperature of the tumors (56.1 °C) in Pd nanosheet-injected mice was higher than that in saline-treated mice (38.7 °C) (Fig. 9C and D). Furthermore, after the *in vivo* photothermal therapy of the tumors in Pd nanosheet-injected mice, the tumors were killed and the mice survived much longer compared to those under the same photothermal treatment in the absence of Pd nanosheets. Besides the Pd nanosheets mentioned above, Au nanoprisms were also found to exhibit an excellent NIR photothermal effect under a longer NIR wavelength of 1064 nm, which is very important as infrared light with a longer wavelength could have greater tissue penetration, owing to its lower optical absorption by blood and soft tissue.^45,134,177^

## 5. Conclusion and outlook

In summary, we briefly described various bottom-up synthetic methods for metal nanoplates and nanosheets and described their unique properties. Importantly, the 2D morphologies and LSPR characters of metal nanoplates/nanosheets make them promising candidates in a range of applications, such as catalysis, SERS, sensing and NIR photothermal therapy. However, there are still some challenges that need to be addressed in order to fully realize the real applications of metal nanoplates and nanosheets.

On the one hand, the sharp corners and edges of metal nanoplates and nanosheets, which play a key role in their excellent properties and various applications, have relatively high surface energies. As a result, they are prone to deformation, degradation or etching under harsh experimental conditions. Although the current solutions, such as coating a thin layer of another metal over the initial metal nanoplates and nanosheets to obtain bimetallic nanostructures, can somewhat improve the stability of metal nanoplates and nanosheets, more effective methods are required to further enhance their chemical and thermal stabilities. As the sharp corners and edges of thin metal nanoplates and nanosheets are critically important, one may consider sculpting their edges and basal surfaces to fabricate wavy edges and mesh-rich basal surfaces, which may further enhance their activities in various applications.

On the other hand, the wide applications of nanoplates and nanosheets of metals, especially noble metals, are limited by their scarce reserves on earth. Therefore, one of the future research directions lies in the preparation of noble metal-based bimetallic, trimetallic and even multimetallic nanoplates and nanosheets with the addition of earth-abundant transition metals, thus decreasing the amount of noble metals involved. Simultaneously, enhanced properties (*e.g.* catalytic properties) and chemical and thermal stabilities from bimetallic, trimetallic and multimetallic nanoplates and nanosheets are expected because the surface lattice strain can induce a shift of the d-band center of the noble metal component.^178^ Moreover, the hybridization of metal nanoplates and nanosheets with other cheap and abundant 2D nanomaterials, such as graphene and transition metal dichalcogenide nanosheets, to fabricate functional composites, is also an effective route to reduce the amount of noble metals used. As is already known, graphene and transition metal dichalcogenide nanosheets can help to stabilize the metal nanostructures to avoid their performance degradation in real applications.^21^ In addition, the interactions between metal nanostructures and graphene or transition metal dichalcogenide nanosheets may further boost the activities of metal nanostructures.^179,180^

The surface areas of metal nanoplates and nanosheets is another critical factor for many applications, especially for catalysis. Currently, the thicknesses of metal nanoplates and nanosheets remain mostly larger than 1.5 nm, which results in relatively small surface areas and thus limits their catalytic activities.^30,31,37,59^ Very recently, the free-standing single-atom layered Rh nanosheet has been successfully synthesized.^27^ Owing to its single-atom layer feature, the proportion of surface Rh atoms in the Rh nanosheets is 100%, and it exhibited excellent catalytic activity in organic reaction catalysis. However, the synthesis of other single-atom layered metal nanostructures has not been achieved yet. Therefore, one of the challenges lies in the controlled synthesis of metal nanostructures with desired thicknesses (*e.g.* atomic thicknesses), shapes and sizes, thereby finely tuning their properties for a wide range of applications with superior performances.
